# Ultrasound-guided kidney biopsy: a ten-year retrospective single-center experience and the promising role of clinical hypnosis

**DOI:** 10.1007/s11255-024-04196-1

**Published:** 2024-09-06

**Authors:** Andrea Angioi, Giacomo Mascia, Danilo Sirigu, Riccardo Cao, Paola Bianco, Daniela Onnis, Matteo Floris, Gianfranca Cabiddu, Antonello Pani, Nicola Lepori

**Affiliations:** 1 Nephrology, Dialysis and Transplantation Unit, Giuseppe Brotzu’ Hospital, Cagliari, Italy; 2 Department of Medical Science and Public Health, University of Cagliari, Cagliari, Italy; 3 Pathology Department, Giuseppe Brotzu’ Hospital, Cagliari, Italy

**Keywords:** Hypnosis, Kidney biopsy, Fine needle biopsy, Glomerular diseases, Ultrasound

## Abstract

This retrospective analysis investigates the outcomes and complications of 682 kidney biopsies performed at ARNAS G. Brotzu from 2010 to 2021. Our findings indicate a minor complication rate of 9.1%, with severe complications being exceedingly rare at 0.3%. Age did not contribute to an increased risk, underscoring the procedure’s safety across age groups. Clinical hypnosis was incorporated into the biopsy protocol in a subset of patients (*n* = 45) from April 2019 to December 2023. Over 90% of these patients reported no perception of the procedure, and 60% experienced no pain. According to STAY-Y test scores, this approach significantly reduced anxiety post-procedure (p = 0.001); no major or minor complications were observed in this group. While our study reaffirms the very low risk of severe complications in kidney biopsies, it also highlights the potential benefits of adjunct clinical hypnosis in enhancing patient comfort and cooperation during the procedure. This exploration opens a promising avenue for further investigation to improve patient experiences and procedural outcomes in kidney biopsies.

**Supplementary Information:**

The online version contains supplementary material available at 10.1007/s11255-024-04196-1.

## Introduction

Ultrasound-guided percutaneous kidney biopsy (USKB) is the cornerstone diagnostic technique for primary and secondary nephropathies. Thanks to its simplicity and safety, it has evolved into an irreplaceable instrument for discerning the pathologic mechanisms of kidney diseases since its first description by Berlyne in 1961 [1].

Kidney biopsy is widely regarded as a safe procedure with a low incidence of severe complications: recent studies suggest that significant complications occur in 0.2–6.6% of patients, while the risk escalates for those admitted to intensive care units, with complications reported in 13–22% of these cases [2, 3]. This variability can largely be attributed to differing protocols for identifying complications or definitions of major and minor events across various institutions.

Bleeding is the most prevalent complication of USKB, mainly due to the kidneys’ rich vascularization and retroperitoneal position, which renders compression maneuvers challenging to halt bleeding.

Currently, a non-invasive approach is gaining traction for selected clinical scenarios. However, its clinical validity is contentious, resulting in significant disparity in indications among institutions (e.g., anti-PLA2R positive nephrotic syndrome) [4]. On the flip side, the safety profile of USKB has improved considerably due to advancements in ultrasound and Doppler imaging techniques, coupled with a more comprehensive investigation of hemostasis (e.g., PFA100) [5].

In some Institutions, a day-surgery kidney biopsy program has been implemented, limiting the observation period to 6 h post-biopsy with similar complication rates to those observed in inpatients [6, 7]. This approach has been encouraged by several data published in the Literature: Atwell et al. describe an incidence of bleeding complications of 1.1% ( 6 cases on 1407 renal biopsies), with only four complications occurring > 24 h after renal biopsy [8].

The integration of clinical hypnosis into medical protocols has been recently explored for its potential benefits in managing procedural pain and anxiety. Hypnotic analgesia has been found effective in reducing the consumption of pain medication during invasive procedures without aggravating pain intensity and anxiety and with a small beneficial effect on lowering procedure length [9].

This study aims to (1) determine the incidence and causative factors of minor and major complications related to the USKB technique as implemented in our clinical practice and (2) assess the supplementary role of clinical hypnosis in the current kidney needle biopsy protocol.

## Materials and methods

We conducted a retrospective analysis on a cohort of patients admitted to the Division of Nephrology, Dialysis, and Renal Transplantation of ARNAS G. Brotzu between January 1, 2010, and February 1, 2021. These patients underwent kidney biopsies using ultrasound-guided techniques on a native kidney. In addition, between April 2019 and November 2022, a subset of 45 patients (14 men and 31 women) received an ultrasound-guided kidney needle biopsy paired with clinical hypnosis. Written informed consent was obtained from all patients, while post-procedure experiences were measured using STAY-Y tests in those treated with hypnosis.

Data collected for each patient included comorbidities, clinical nephrological syndromes, first-level (e.g., blood count) and second-level (e.g., serum immunoglobulins) tests, definitive histologic diagnosis, and observed post-procedure complications (Table 1). Patients with incomplete clinical datasets and kidney transplant recipients were excluded from the study.

**Table 1:** Baseline demographic and clinical characteristics of our cohort

Patients (n)	682
Age—years	
Median	55
Interquartile range	29–81
Male sex—no. (%)	388 (57)
Age groups, years (%)	
0–19	21 (3.1)
20–49	247 (36.2)
50–65	203 (29.8)
> 65	211 (30.9)
Italy (Sardinia)—no. (%)	630 (92.4)
> 2 biopsies—no. (%)	74 (10.9)
Bleeding time—seconds	
Median	270
Interquartile range	180–360
PT INR	
Median	1.01
Interquartile range	0.95–1.08
Platelets (105/mm^3^)	
Median	272
Interquartile range	215–343
Hemoglobin (g/dl)	
Median	11.9
Interquartile range	10.1 – 13.6
Serum Creatinine (mg/dl)	
Median	1.44
Interquartile range	0,87–2.65
BUN (mg/dl)	
Median	30
Interquartile range	19.5–51.0
Serum albumin (g/dl)	
Mean (± SD)	2.9 (± 0.9)
24 h proteinuria (g)	
Median	2.97
Interquartile range	1.02–5.78
Hypertension—no. (%)	405 (59)
Diabetes—no. (%)	95 (13.9)
Paraproteinemia—no. (%)	62.1 (9.1)
Infectious diseases—no. (%)	48 (7)
Solid tumors—no. (%)	14 (2.1)
Liver disease—no. (%)	45 (6.6)

We employed 16-gage needles until 2018, after which we switched to 18-gage needles*.* A trained specialist administered clinical hypnosis. The biopsy was performed only after confirming normal coagulation parameters (PT INR < 1.2; PTT < 35 s; FBG > 150 mg/dl) and adequate platelet count (> 100,000/mm^3^). Medications interfering with platelet function were held ten days before the procedure, while anticoagulation agent discontinuation was managed according to available guidelines [10]. All the procedures were performed on hospitalized patients. After the procedure, patients were closely monitored to assess complications promptly: blood pressure and vital signs were frequently checked, urine was monitored seeking for macroscopic hematuria, and a complete blood count was performed 4- and 8 h after the renal biopsy. New onset lumbar pain was promptly evaluated by nephrologists trained in ultrasound imaging. After 48 h from a kidney biopsy, patients were discharged if other reasons for hospitalization were absent. Before discharge, all patients underwent a renal ultrasound to assess the presence of perirenal hematoma or AV fistulas.

Post-procedure complications were categorized according to their potential threat to life into minor (e.g., localized hematoma, macrohematuria without the need for a blood transfusion, vegetative symptoms, arteriovenous fistula), and severe (bleeding requiring blood transfusion, the necessity for surgery, nephrectomy, interventional radiology) events.

### Clinical hypnosis protocol

The protocol used for hypnosis at our Center is performed by a radiologist with long experience in ultrasound-guided biopsies and skills in clinical hypnosis. The protocol has three phases.

#### Phase 1

We started equipping the patient on the operating table with high-resolution audio headphones that allow them to listen to the hypnotist, fitted with a microphone and music. The relaxation induced by music is the prelude to the verbalized phase, during which the patient is invited to welcome the environment and to take a comfortable posture, facilitating his body and mental relaxation.

#### Phase 2

Relaxation reduces stress, anxiety, and fear, facilitating empowerment. Using a regressive numeric count improves internal focus, promoting verbalized relaxation suggestions. The patient gradually increases his internal focus through attention to his breath and different body districts. At first, the patient’s concentration is focused on his head and then, in sequence, downwards to his feet. In this way, the association of the regressive numeric count from 10 to 1 implies going deeper and deeper into the hypnotic experience.

#### Phase 3

We induce the patient to bring his attention to a “Safe Place.” We performed this process using suggestions without specific content but capable of diverting the subject’s attention from a surgical environment to a virtual reality. This new “safe place” evoked by patient mental images has the features of an authentic experience in a general condition of well-being, strangeness, and security. When the patient reaches a deep hypnotic state, we start the procedure.

Statistical analyses were performed using both descriptive and inferential statistics. The Shapiro–Wilk and Anderson–Darling tests were used to evaluate data normality. Mean and standard deviation were utilized for normally distributed data, while median and interquartile range were used for non-normally distributed data. A univariate analysis was carried out to ascertain the impact of various clinical variables on the risk of developing complications.

## Results

According to inclusion criteria, kidney biopsy was performed on 682 individuals (Table 1). The median age of the patients was 55.0 years [IQ 29–81]. Most of the procedures were performed in the 20–49-year span (247 cases, equal to 36.2% of the total), followed by the > 65-year age group (211 patients, 30.9%) and the 50–65 age group (203 cases, equal to 29.8%). According to some Expert opinions and recent guidelines, 9.7% (66) of patients were defined as “elderly” (> 75 years), and 4.4% (30) as “very elderly (> 80 years). Male sex was prevalent, comprising 57% of individuals. The patients were predominantly Caucasians (97.5%), 95% were Italian (92.4% from our regional area), and 3.4% (23) were of non-Italian nationality.

In 10 years of retrospective investigation, 89.1% of patients performed a single procedure, 10.3% underwent two, and 0.6% three. The clinical nephrological syndrome of onset was mainly accompanied by rheumatic or autoimmune diseases (20%), arterial hypertension (59%), type II diabetes mellitus (13.9%), smoking (9%), liver disease (6.6%), paraproteinemia (9.1%).

The indication for kidney biopsy was based on the clinical nephrological syndrome of onset. Nephrotic syndrome was the most frequent indication (45%), followed by nephritic syndrome (26%) and urinary abnormalities (25.4%). Moreover, 52.8% of patients showed kidney failure at the time of kidney biopsy (which does not exclude coexistence with those above clinical nephrological syndromes). Of this group, 40.6% of patients had clinical/instrumental characteristics of chronic kidney disease, 11.3% of acute kidney failure, 10.0% acute over chronic kidney failure, and 6.3% rapidly progressing kidney failure.

Upon entering the ward, 93% of patients had hemoglobin values ​​compatible with a safe procedure execution; all outliers were normalized with the initiation of therapy with erythropoietin stimulating factors (ESA) or blood transfusion. 96% of patients had normal PT INR values ​​upon entering the ward; outliers were corrected with oral vitamin-K supplementation. Up to 98% of patients showed a normal bleeding time upon entering the ward; outliers were treated with desmopressin (4 mg/fl/10 kg body weight, up to a maximum of 8 fl) until the bleeding time was corrected before the procedure (Table 1). Desmopressin was also used for those patients with normal bleeding time who showed a moderate–severe reduction in renal function (serum creatinine > 2 mg/dl).

We found 62 intra or post-procedural complications out of 682 needle biopsies (9.1%). Major complications showed an incidence of 1% (7 patients); only three (0.3%) presented a hemorrhage that needed an interventional approach (three required an angiographic approach, and two were unresponsive and underwent a radical nephrectomy). The first patient who underwent a radical nephrectomy was a young anorexic female patient who underwent renal biopsy for AKI in the context of abuse of phosphate-containing laxatives: it is plausible that frailty due to severe malnutrition played a crucial role in developing of major complication. The second case of radical nephrectomy was related to a rupture of the left adrenal artery in a young female patient with severe SLE and AKI due to lupus nephritis: bleeding started eight days after the procedure, making unlikely a direct role of renal biopsy in bleeding. Four patients (0.6%) required a blood transfusion, while 50 (7.3%) had hematomas of the kidney lodge without transfusion. One patient (0.1%) developed an artero-venous fistula (0.2%) that, in one case, required an interventional approach. Of note, two patients (0.3%) had a urinary obstruction due to macroscopic hematuria and bladder clots, thus requiring mechanical thrombectomy and cystoclisis. All bleeding complications needed for blood transfusion were observed within 48 h of hospitalization, except for one patient: no deaths were reported (Table 2).

**Table 2:** Incidence and types of complications in the study cohort, with events classified as major or minor complications

Event	*N (%)*
No complications	621 (91.1)
Major complications	7 (1.0)
Hemorrhage requiring blood transfusion	4 (0.6)
Arteriovenous fistula requiring intervention	1 (0.1)
Nephrectomy	2 (0.3)
Minor complications	54 (7.0)
Hemorrhage not requiring a blood transfusion	50 (7.3)
Acute urine retention	2 (0.3)
Arteriovenous fistula not requiring intervention	1 (0.1)
Lipotimia	1 (0.1)

The univariate analysis excluded a clinical variable influencing the risk of developing an intra or post-procedural complication. Anemia, platelet count, bleeding time, and PT INR were not significantly associated with adverse events since they were clinically corrected before the procedure. Age was not a complication-risk factor, remarking the procedure’s safety in elderly patients (Table 3).

**Table 3:** Role of independent variables predicting every complication (logistic regression with significant variables on the univariate analysis)

Independent variable	Coeff (B)	S.E	Exp(B)	95% C.I. for EXP(B)		Sig
				Lower	Upper	
Bleeding time	– .004	.012	.996	.972	1011	.769
PT INR	5.815	8142	335.3	.000	155.621	.475
Platelets	.000	.000	1.000	1.000	1000	.694
Hemoglobin	– .884	.994	.413	.059	1135	.374
Serum creatinine	– .037	.259	.963	.580	1139	.885
Proteinuria	– .084	.267	.920	.545	1076	.754
Age	.101	.104	1.107	.903	1006	.330
*Diabetes [1]	.785	1.656	2.192	.085	3726	.636
IgG	– .001	.001	.999	.997	1000	.636
C3	– .013	.030	.987	.931	1029	.651
C4	.004	.032	1.004	.943	1019	.908

^*^Hypertension and Diabetes showed collinearity; thus, only diabetes was considered

Hypnosis. From April 2019 to November 2022, 45 patients (20 men and 25 women) admitted to the ARNAS G. Brotzu Hospital underwent ultrasound-guided kidney needle biopsy with the adjunct of clinical hypnosis. Participation in the clinical hypnosis protocol was voluntary. Post-procedure evaluations revealed a significant reduction in anxiety levels compared to pre-biopsy assessments, as measured by the STAY-Y test (p = 0.001). Notably, 92.5% of patients reported no vegetative symptoms and did not perceive the procedure itself. Furthermore, 60% of patients reported no pain during the biopsy on a scale ranging from no pain [1] to severe pain [4], with only one patient (2.2%) experiencing moderate pain during the procedure. All patients expressed a sense of well-being following the procedure. Specifically, on a scale ranging from very anxious [1] to very calm [4], 58% of patients reported feeling calm, and 33% reported feeling very calm during hypnotic sedation, underscoring the effectiveness of hypnosis in managing anxiety. In addition, 14% of respondents had no recollection of the procedure or their level of participation. Importantly, no major or minor complications were observed in the cohort undergoing clinical hypnosis.

## Discussion

This decade-long retrospective review delineated a relatively low incidence of significant complications following kidney biopsies, demonstrating 62 intra or post-procedural complications from 682 needle biopsies (9.1%)​; only 1% were severe. A similar incidence of major complications has been described in a recent multicentric Italian study, with a reported incidence of 1.1% for red blood cell transfusion and 0.5% for invasive post-biopsy procedures[11]. Our results are in the context of the heterogeneous data available in Literature, which report an incidence of complications of 0.2–11% [2, 6]. The significant heterogeneity of the data can be attributed to different factors, primarily the different types of studies published in Literature: the highest rate of major complication has been described in population-based studies (incidence of 26% for blood transfusion in the study from Al Turk et al.) several confounding factors may explain the high rate of complications, which is not entirely attributable to the biopsy procedure [12]. Second, the different policies of each Center in post-biopsy surveillance could explain variability in reported complications. Data from groups that routinely perform post-biopsy ultrasound monitoring show a higher incidence of minor complications (mainly asymptomatic hematomas) than observed in data published by those nephrological Centers in which ultrasound monitoring is performed based on clinical indications (e.g., new onset of pain, gross hematuria, drop in serum hemoglobin, hypotension). Lastly, a higher incidence of major complications can be attributed to the patient’s different pre-biopsy assessments. In 2018, Antunes et al. reported an incidence of major complications of 11% in their cohort of 238 patients who underwent renal biopsy. Interestingly, according to their center policy, the biopsy was performed even in patients with a low platelet count (> 60.000/mm^3^) [13]. The rigorous selection policy of renal biopsy candidates at our Center (platelets > 100,000/ mm^3^, optimal blood-pressure control, Hb > 8 g/dl, normal bleeding time) could, therefore, be one of the elements underlying the low incidence of major complications observed in our cohort. This empirical evidence bolsters the argument that when administered under suitable conditions and by experienced professionals, kidney biopsy is primarily a safe procedure with a marginal risk of complications.

The univariate analysis could not detect any clinical variables substantially contributing to the risk of intra or post-procedural complications​​. Previously published data show the presence of several risk factors associated with an increased incidence of major complications. Data published in 2014 by the Rush University Chicago group from a large cohort of 1055 patients show a sevenfold increase in the risk of major complications in those patients with systolic BP values > 170 mmHg and a threefold increase in patients with Hb values < 11 g/dl [14]. Consistent with these data, Schorr et al. proposed a predictive model for the occurrence of major complications following renal biopsy in 2020: the combination of age, platelet count, pre-procedure hemoglobin, low BMI, and small kidney size were the variables correlated with determining risk [15]. Another exciting aspect, still debated in the literature, is the correlation between the size of the needle used for the procedure and the risk of major complications. Antunes et al. showed a higher incidence of major complications with the use of needle size 16 G than with needle size 18 G (OR 5.1). Although using 16-G needles was associated with more glomeruli in the biopsy sample, the diagnostic power was not higher in this group of patients [13]. In contrast to the results published by Antunes et al., Sousanieh’s 2020 results did not show a significant correlation between needle size and risk of major complications [16]. The results of the meta-analysis published in 2020 by Poggio et al. support the absence of a correlation between needle size and risk of complications; the authors concluded their analysis by arguing that using a 16-G needle size ensures adequate samples with an acceptable safety profile [17].

The absence of clear evidence of risk factors for major complications observed in our Cohort, consistent with the low incidence of complications, suggests that meticulous patient selection and comprehensive pre-procedural preparation, encompassing the correction of anemia, platelet count, and PT INR, are critical determinants for ensuring the safety of kidney biopsies [7]. Our findings also emphasize the non-significant impact of age on the risk of complications, thereby underscoring procedural safety even for the elderly demographic.

### Role of clinical hypnosis in kidney biopsy

The American Psychological Association defines hypnosis as a state of modified consciousness involving focused attention and reduced peripheral awareness characterized by an enhanced capacity for response to suggestion [18]. Hypnosis associates a set of techniques that can be used independently of each other, making this tool a multifaceted therapy (e.g., hypno-analgesia for the management of pain, hypno-sedation primarily used in anesthesia, and hypnotherapy for psychotherapeutic applications). In a 2015 report from the French National Institute of Health and Medical Research (INSERM), the effectiveness of hypnosis has been demonstrated for reducing the consumption of analgesics or sedatives during invasive procedures, in surgery, or in interventional radiology [19]. Hypnotic analgesia has been tested in many trials to reduce pain and anxiety during both medical and surgical procedures [20]. Invasive procedures in which hypnotic analgesia has been used together with usual pain medication with greater effectiveness compared to conventional care or standard pain medication include large core breast biopsy, percutaneous tumor treatment, radiologic, percutaneous vascular, and cardiovascular [21]. Besides ameliorating pain, the consumption of pain medication was, in some studies, reduced. In addition, the procedure lengths and the number of adverse events were decreased in several studies [22]. A recent meta-analysis published in 2019 by Noergaard et al. showed hypnotic analgesia was effective in reducing consumption of analgesics: a slight effect was found on experienced anxiety and pain intensity [9]. Clinical hypnosis’s role in renal biopsy has been evaluated in only one study published in 1984 in a pediatric setting. Forty-five children were randomized to receive or not clinical hypnosis before renal biopsy. The results discussed by the authors showed a sharp reduction in the execution times of the biopsy procedure: data relating to intra- and post-procedural pain control and those relating to the incidence of complications were not reported. Our study is, to the best of our knowledge, the first report of the use of clinical hypnosis in kidney biopsy in adult patients.

Moreover, patients who underwent clinical hypnosis were evaluated for incidence of complication, and post-procedure experiences were assessed with a validated questionnaire. Our results highlight the beneficial role of hypnosis in effectively attenuating anxiety and discomfort among patients undergoing ultrasound-guided renal needle biopsies. These observations correspond with the existing body of research that advocates for the merits of hypnosis in managing pain and anxiety during invasive procedures​. Remarkably, 92.5% of patients under hypnosis reported no vegetative symptoms and a lack of procedure awareness. The application of hypnosis additionally ensured optimal tissue sampling during biopsies, obviating the necessity for procedure repetition. Furthermore, adopting hypnosis seems to enhance patient cooperation, allowing the kidney biopsy in patients in whom the anxious state would have made it impossible to carry out the examination or, at the very least, would have required a pharmacologic-sedation protocol.

Despite these findings, our study bears several limitations that merit consideration. First, as a descriptive analysis without randomization or control group comparisons, the study’s design inherently limits the ability to draw definitive causal inferences and may introduce biases. Second, the retrospective nature of the study could introduce selection bias. Thirdly, the single-center setting may not encompass the heterogeneity in practices and patient populations encountered across diverse healthcare contexts..

To summarize, our retrospective experience supports kidney biopsies’ safety profile, signifying a modest incidence of serious complications. Thorough patient selection and pre-procedural preparation, including correcting anemia, platelet count, and PT INR, are indispensable for securing the safety of kidney biopsies. Moreover, our experience underlines the role of adequate skilling in renal biopsy technique and the role of a well-defined monitoring protocol, emphasizing the need to centralize this procedure in Centers with high experience. Incorporating hypnosis as a supplemental approach to ultrasound-guided renal needle biopsy is a promising strategy for alleviating anxiety and pain, potentially amplifying patient cooperation and overall procedural success..

## Supplementary Information

Below is the link to the electronic supplementary material.

Supplementary file1 (DOCX 18 KB)

## Data Availability

No datasets were generated or analysed during the current study.
