# Supplementary material for: Ultrasound-guided kidney biopsy: a ten-year retrospective single-center experience and the promising role of clinical hypnosis
Source: Int Urol Nephrol. 2024 Sep 6;57(2):553–9. doi: 10.1007/s11255-024-04196-1 (PMC11772539; doi:10.1007/s11255-024-04196-1)
Supplement: Supplementary file 1 — Supplementary file1 (DOCX 18 KB) [file 11255_2024_4196_MOESM1_ESM.docx]

**Supplementary Table 1.**

| Patients (n) | Hypnosis |
| --- | --- |
|  | 45 |
| Age - years |  |
| Median | 44.5 |
| Interquartile range | 15-74 |
| Male sex – no. (%) | 20 (44.4) |
| Age groups, years (%) |  |
| 0-19 | 4.2 |
| 20-49 | 58.3 |
| 50-65 | 20.8 |
| >65 | 16.7 |
| Italy (Sardinia) – no. (%) | 45 (100) |
| >2 biopsies – no. (%) | 4 (8.8) |
| Bleeding time - seconds |  |
| Median | 330 |
| Interquartile range | 270-390 |
| PT INR |  |
| Median | 1.01 |
| Interquartile range | 0.98-1.09 |
| Platelets (105/mm3) |  |
| Median | 257 |
| Interquartile range | 219 - 342 |
| Hemoglobin (g/dl) |  |
| Median | 11.0 |
| Interquartile range | 9.4 – 11.4 |
| Serum Creatinine (mg/dl) |  |
| Median | 1.53 |
| Interquartile range | 0.78 – 2.21 |
| BUN (mg/dl) |  |
| Median | 29.5 |
| Interquartile range | 19.2 – 49.0 |
| Serum albumin (g/dl) |  |
| Mean (±SD) | 3.0 (± 0.8) |
| 24h Proteinuria (g) |  |
| Median | 1.28 |
| Interquartile range | 0.40 – 4.37 |
| Hypertension – no. (%) | 11 (45.8) |
| Diabetes – no. (%) | 4 (16.7) |
| Infectious diseases – no. (%) | 2 (8.3) |
| Solid tumors – no. (%) | 0 (0) |
| Liver disease – no. (%) | 1 (4.2) |

Supplementary table 2.

|  | *Total* | *Hypnosis* |
| --- | --- | --- |
| *Event* | *N (%)* | *N (%)* |
| **No complications** | **621 (91.1)** | **45 (100)** |
| **Major complications** | **7 (1.0)** | 0 (0.0%) |
| Hemorrhage requiring blood transfusion | 4 (0.6) | 0 (0.0%) |
| Arteriovenous fistula requiring intervention | 1 (0.1) | 0 (0.0%) |
| Nephrectomy | 2 (0.3) | 0 (0.0%) |
| **Minor complications** | **54 (7.0)** | 0 (0.0%) |
| Hemorrhage not requiring blood transfusion | 50 (7.3) | 0 (0.0%) |
| Acute urine retention | 2 (0.3) | 0 (0.0%) |
| Arteriovenous fistula not requiring intervention | 1 (0.1) | 0 (0.0%) |
| Lipotomy | 1 (0.1) | 0 (0.0%) |
